# Investigation of the Fluorescence Turn-off Mechanism, Genome, Molecular Docking *In Silico* and *In Vitro* Studies of 2-Acetyl-3*H*-benzo[*f*]chromen-3-one

**DOI:** 10.1021/acsomega.2c02424

**Published:** 2022-06-29

**Authors:** Varsha V. Koppal, Raveendra Melavanki, Raviraj Kusanur, Zabin K. Bagewadi, Deepak A. Yaraguppi, Sanjay H. Deshpande, Ninganagouda R. Patil

**Affiliations:** † Department of Physics, KLE Technological University, Hubli 580031, Karnataka, India; ‡ Department of Physics, M S Ramaiah Institute of Technology, Bangalore 560054, Karnataka, India; § Department of Chemistry, RV College of Engineering, Bangalore 560059, Karnataka, India; ∥ Department of Biotechnology, KLE Technological University, Hubli 580031, Karnataka, India; ⊥ Department of Physics, B V B College of Engineering and Technology, Hubli 580031, Karnataka, India

## Abstract

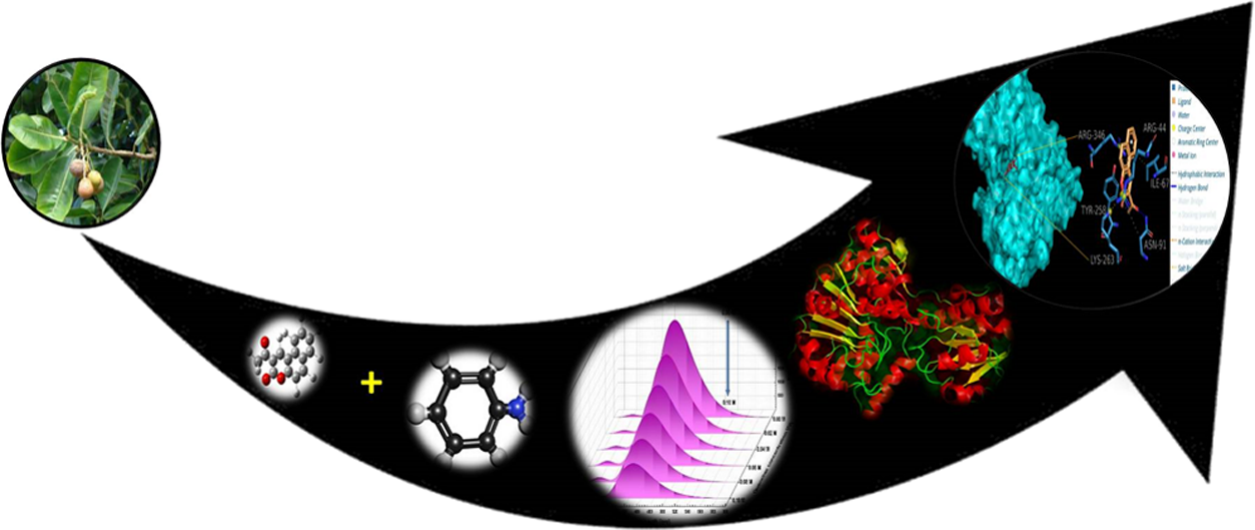

The present study harnesses fluorescence quenching between a nonfluorescent aniline and fluorophore 2-acetyl-3*H*-benzo[*f*]chromen-3-one [2AHBC] in binary solvent mixtures of acetonitrile and 1,4-dioxane at room temperature and explores the fluorophore as an antimicrobial material. Our findings throw light on the key performance of organic molecules in the medicinal and pharmaceutical fields, which are considered as the most leading drives in therapeutic applications. In view of that, fluorescence quenching data have been interpreted by various quenching models. This demonstrates that the sphere of action holds very well in the present work and also confirms the presence of static quenching reactions. Additionally, the fluorophore was first investigated for druglike activity with the help of *in silico* tools, and then it was investigated for antimicrobial activity through bioinformatics tools, which has shown promising insights.

## 1 Introduction

The fluorescence quenching mechanism has become an imperative spectroscopic tool to investigate biophysical and biochemical systems in different liquid media. It reveals important information about proteins, membranes, and macromolecular assemblies. For fluorescence quenching studies, we generally select quenchers such as carbon tetrachloride, aniline, bromobenzene, various metal ions, and quantum dots. The selection of quencher for the respective fluorophore mainly depends on the suitability of the molecular electronic structure of the quencher with the fluorophore.^1,2^ Recently, there has been an increased interest in photochemistry and photobiology among scientists and researchers because of the potential applications of fluorescence quenching in the field of molecular dynamics and molecular imaging. The fluorescence quenching study helps in understanding the dynamic changes of proteins in complex macromolecular systems to navigate microbial cell growth, *etc*.^3−7^ The main reason for fluorescence quenching is various molecular interactions such as energy relocation, molecular reorganization, collisional quenching, and ground-state complex formation.^8^ However, quenching mechanisms are also affected by the type of the quencher and the nature of the fluorophores.^9^ Fluorescence quenching can be employed to study protein folding at the single-molecule level,^10^ and further is constructive to acquire information about the conformational and/or dynamic changes of proteins in complex macromolecular systems.^11^

Coumarins have been the most important compounds for photophysical studies during the last few decades, as they are exceedingly fluorescent in nature. Coumarin is a plant-derived product that is naturally available in different plants such as cassia cinnamon, tonka beans, strawberries, apricots, black currents, *etc*.^12^ They are the most widely used organic substances in therapeutic applications as photochemotherapy, antibacterial, anti-inflammatory, antitumor, and anti-HIV therapy agents, central nervous system (CNS) stimulants, and anticoagulants and dyes. A recent study on coumarin shows that coumarin-containing drugs can decrease estrogenic activity, which arises during menopause.^13,14^ Over the past few decades, technological improvements in both optics and electronics have greatly expanded fluorometric applications, predominantly in analytical fields, as a consequence of the high sensitivity and specificity afforded by the techniques. Using fluorometry in the study and conservation of cultural heritage is a recent development.^15^ The recent outburst in new fluorescence applications is accelerating the rapidity of research and development in basic and applied life sciences, including genomics, proteomics, bioengineering, medical diagnosis, and industrial microbiology. Fluorescence-based techniques are extensively used to address fundamental and applied questions in the biological and biomedical sciences. The current use of fluorescence-based technology includes assays for biomolecules, metabolic enzymes, DNA sequencing, research into biomolecule cell signaling dynamics and adaptation, and fluorescence *in situ* hybridization (FISH) to identify specific DNA and/or RNA sequences in tissues. Recently, molecular methods have been applied to fuse the gene for the green fluorescent protein (GFP) to other genes, leading to its expression in living cells. This allows a sophisticated breakdown of gene expression and cellular location of important structural proteins and enzymes. Extreme selectivity of fluorescent labels that can target specific organisms opens new avenues to resolve industrially and medically relevant problems in areas such as public health, the safety of foods, and environmental monitoring. Innovative fluorophores, new techniques including spectrally and temporally resolved fluorescence, and purpose-engineered instrumentation create niche commercial opportunities and lead toward tangible industrial outcomes. Fluorescence spectroscopy is one of the most sensitive and versatile instruments in medical, biological, and biochemical research. Table S1 lists some of its numerous applications.^16^

The fluorescence quenching of coumarins provides beneficial information about structural and dynamic properties. Studies have shown that effectively adding a substituent group to the parent molecule influences and enhances the biochemical and pharmacological properties.^17−19^ Coumarins have shown significant growth in the use of fluorescence in biological sciences, especially in biochemistry and biophysics, along with environmental monitoring, clinical chemistry, DNA sequencing, and genetic analysis by fluorescence *in situ* hybridization. Especially in molecular biology, fluorescence is used for cell identification and sorting in flow cytometry and in cellular imaging to reveal the localization and movement of intracellular substances by means of fluorescence microscopy. Because of the high sensitivity of fluorescence detection, there is continuing development of medical tests based on the phenomenon of fluorescence. These tests include widely used enzyme-linked immunoassays and fluorescence polarization immunoassays.

In the literature, only few articles are focused on fluorescence quenching of coumarin compounds in binary solvent mixtures, ADMET, molecular docking, *in silico* and antimicrobial studies. Hence, the above insights have enlightened us to undertake a detailed analysis on the title fluorophore. This present study would help us to find the possible applications of 2AHBC for molecular imaging and biomedical applications.

## 2 Experimental Section

### 2.1 Materials

The fluorescent probe 2-acetyl-3*H*-benzo[*f*]chromen-3-one [2AHBC] was freshly synthesized as reported in the literature.^20^ Aniline is used as a quencher. Acetonitrile (ACN) and 1,4-dioxane (DXN) solvents are selected to prepare binary solvent mixtures. The solubility of the solute was checked prior to conducting the experiment. The solvents ACN, DXN, and quencher aniline were procured from S-D Fine Chemicals Ltd., India; they were of HPLC grade and used as received. To minimize self-quenching and reabsorption, the concentration of solute was kept minimum (1 × 10^–6^ M) in all of the solvent mixtures. Preparing a fresh solution prior to conducting the experiment would help in getting good results. The aniline concentration varied from low 0.00 M to high 0.01 M in all of the binary mixtures.

### 2.2 Absorption, Emission, and Lifetime Measurements

The study of spectroscopic properties was carried out in various concentrations of ACN + DXN mixture by measuring absorption spectra using a UV/vis spectrophotometer (model: U-3300) with a wavelength accuracy of 0.5 nm and emission spectra using a Hitachi spectrophotometer F-7000 at room temperature. For every measurement, the spectrometer was calibrated for the baseline. All of the glassware were cleaned well prior to preparing the solutions. Fluorescence lifetime measurements were carried out for the 2AHBC molecule with and without a quencher using a spectrometer (model: ChronosBH, TCSPC). All of the results obtained from the experiments can be reproducible within 5% of the experimental error.

### 2.3 Selection of Target

*Pseudomonas aeruginosa* is a facultative pathogen, which has very high medical importance because of its resistant nature. *P. aeruginosa* is a multi-drug-resistant pathogen because of its ubiquitous nature. Intrinsic mechanisms that are responsible for the resistance have a great impact on the illness caused.^21^ A number of therapeutic targets to target *P. aeruginosa* have already been studied, but it is very important to select a target that is part of the antibiotic resistance mechanism. Targeting proteins involved in antibiotic resistance not only helps in reducing resistance but also helps in controlling the growth of pathogens.^21^ To find a suitable target, the complete genome of *P. aeruginosa* was downloaded with assembly idGCF_000006765.1 from NCBI Genome. The downloaded genome was submitted to the ARTS tool for the prediction of essential (core) genes and resistant gene models.^22^

### 2.4 ADMET, Drug-Likeness, and Molecular Docking

Adsorption, distribution, metabolism excretion, and toxicity analysis (ADMET) is one of the valuable processes present in the discovery pipeline of drugs. The main aim of ADMET studies is to find the physiochemical properties of the compound, which are a very important part of compound design and optimization. Oral absorption, receptor toxicity, clearance, and brain dispersion are significant features to be observed in ADMET studies.

Molecular docking is performed to calculate the binding affinity and the best possible mode of the ligand in a complex with protein.^23,24^ The structure of 2-acetyl-3*H*-benzo[*f*]chromen-3-one was obtained from the PubChem database (Pubchem id: 748172),^25^ and the structure was optimized using Open Babel^26^ with minimization steps of 5000; the weighted rotor search method was employed to generate 50 conformations for the compound, and minimization was carried out using the steepest descent method.^27^ In the current study, Erythronate-4-phosphate dehydrogenase was chosen as the target based on the ARTS prediction. The crystal structure of the protein was downloaded from the PDB database^28^ and a reference-based docking study was carried out using Autodock vinatool.^29^ The grid box was generated with the specification of 3.5 Ao areas around the bound cocrystallized ligand.^30^

### 2.5 Antimicrobial Activity of 2-Acetyl-3*H*-benzo[*f*]chromen-3-one

The *In vitro* antibacterial capability of the synthesized compound, 2-acetyl-3*H*-benzo[*f*]chromen-3-one, was evaluated by the agar well diffusion technique according to the method published previously by Yaraguppi et al.^30^ The pathogens were obtained from the Microbial Type Culture Collection and Gene Bank (MTCC), Chandigarh, and the National Collection of Industrial Microorganisms (NCIM), Pune. The antibacterial effect was assessed against Gram-positive bacteria like *Micrococcus luteus* (NCIM 2871), *Bacillus cereus* (NCIM 2217), *Bacillus subtilis* (NCIM 2718), and *Staphylococcus aureus* (MTCC 737). Also, it has been tested against Gram-negative pathogens such as *Escherichia coli* (MTCC 443), *Salmonella typhimurium* (MTCC 98), *P. aeruginosa* (MTCC 2297), and *Klebsiella pneumoniae* (MTCC 109). The pathogenic cultures (24 h broth) were spread-plated on the nutrient agar plates. Wells were created on the inoculated plates with a sterile cork borer for loading the compound. 2-Acetyl-3*H*-benzo[*f*]chromen-3-one (100 μl, 5 mg mL^–1^) prepared in dimethyl sulfoxide (DMSO) was filled in the agar wells and allowed for diffusion. The compound-loaded plates were incubated for 24 h at 35 ± 2 °C. DMSO and gentamicin were used as negative and positive controls, respectively. The zone of inhibition (mm) was recorded. The *in vitro* antifungal property was examined by employing the agar disk diffusion method following the method reported by Bagewadi et al.^31^ The compound was tested against a selected pathogen, *Candida albicans*, which is our lab isolate. The fungal pathogen was grown in potato dextrose broth (24 h) and inoculated on potato dextrose agar plates. The compound was loaded on Whatman filter paper no. 1 disks (6 mm) and kept on the agar plates. The inoculated cultures were incubated for 48 h at 30 ± 2 °C. The inhibition zone (mm) was measured. Positive control was clotrimazole.

### 2.6 Determination of the Minimum Inhibitory Concentration (MIC)

MIC is known as the maximum dilution of the compound that exhibits the inhibition of microbial growth. The MIC was executed as per the method illustrated by Bagewadi et al.^32^ using microdilution technique. The synthesized compound stock (5 mg mL^–1^) was diluted (using DMSO as diluent) to achieve a concentration between 5 and 0.625 mg mL^–1^. The bacterial pathogen *P. aeruginosa* was inoculated in nutrient broth and agitated with the respective concentrations of the compound for 24 h incubation (35 ± 2 °C). The turbidity was assessed by recording OD at 660 nm.

## 3 Results and Discussion

### 3.1 Fluorescence Quenching of 2AHBC in Binary Solvent Mixtures

**Figure 1:**
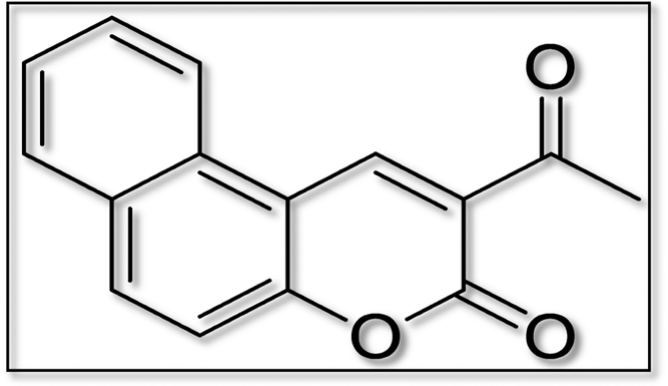
Molecular structure of the 2AHBC molecule.

The molecular structure of 2-acetyl-3*H*-benzo[*f*]chromen-3-one [2AHBC] is presented in Figure 1. Valuable information regarding quencher interactions with the fluorophore can be obtained by combining stationary and time-resolved measurements.

**Figure 2:**
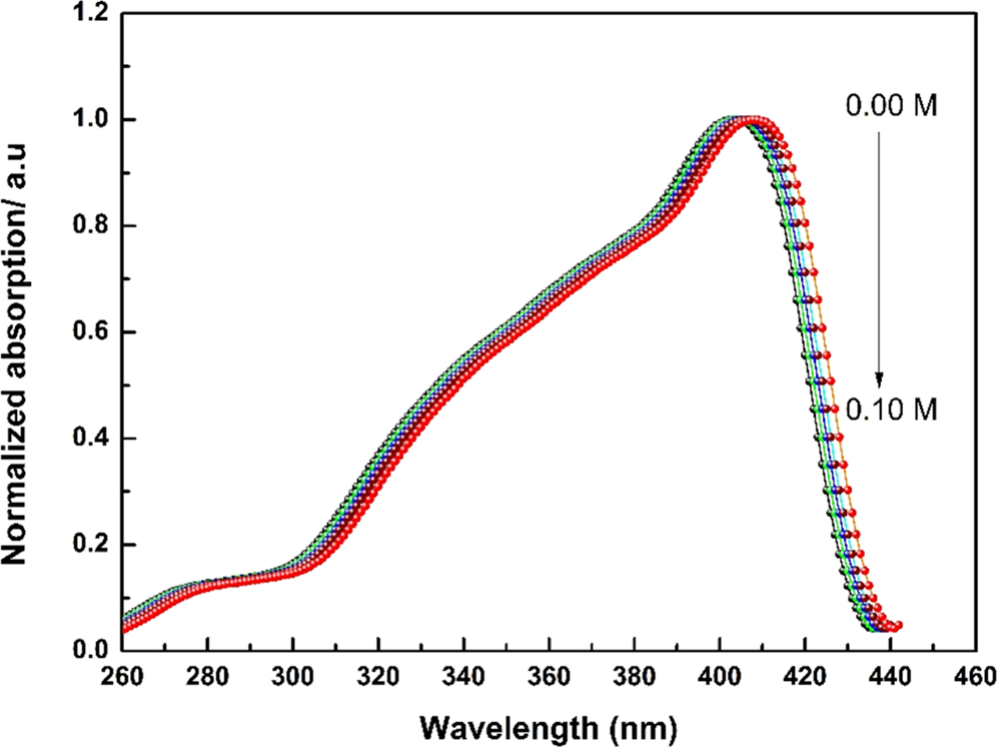
Typical normalized absorption spectrum of 2AHBC + different concentrations of aniline in 60% ACN + 40% DXN solvent mixture.

**Figure 3:**
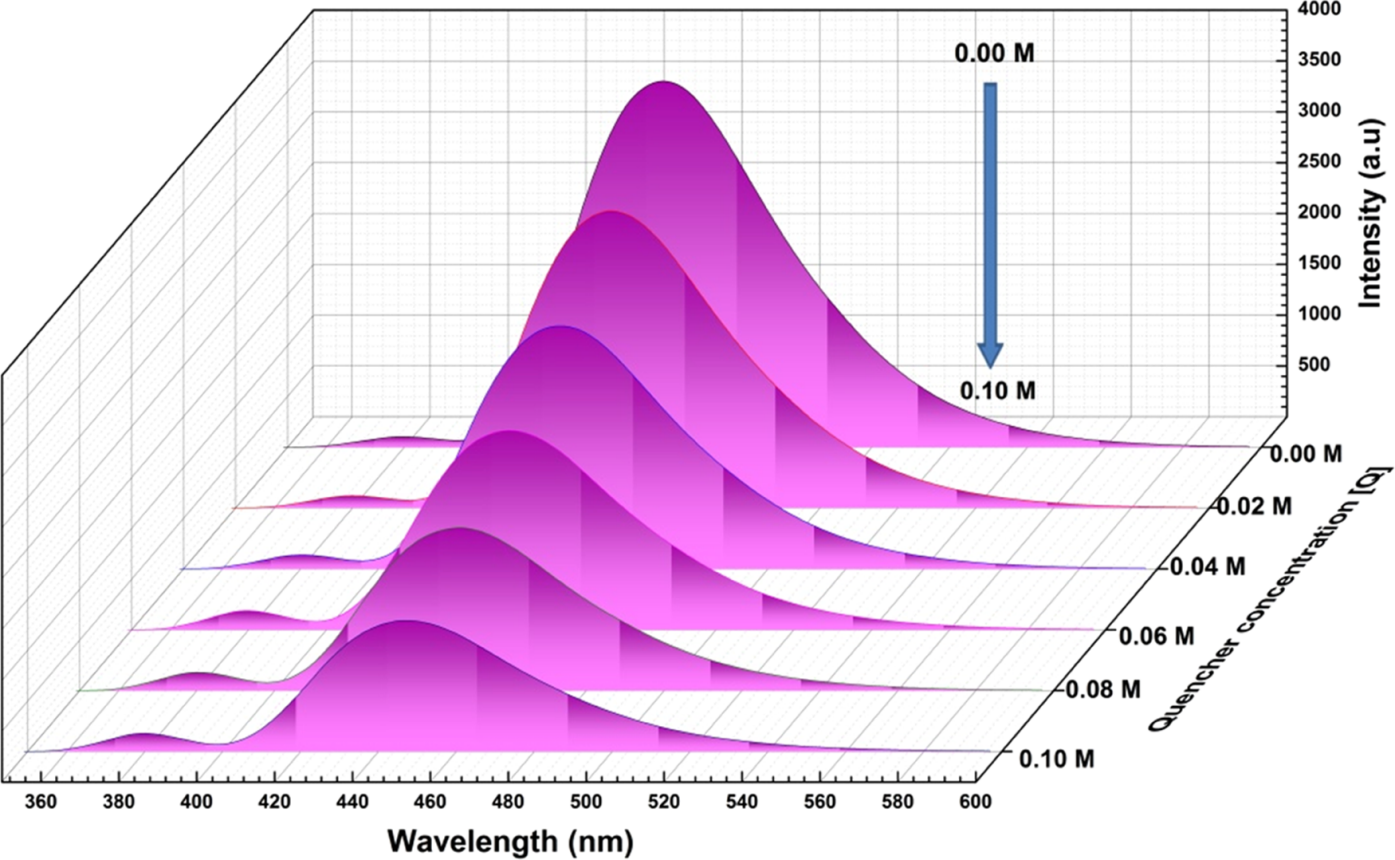
Typical fluorescence spectrum of 2AHBC + different concentrations of aniline in 60% ACN + 40% DXN solvent mixture.

Typical absorption and emission spectra of 2AHBC were recorded with and without aniline at room temperature and are shown in Figures 2 and 3, respectively. A slight bathochromic shift was observed in the absorption spectra (Figure 2) due to the reactive nature of the solute toward the polarity of the solvent mixtures. Table 1 represents the excitation wavelength, emission wavelength, and peak intensity of the fluorophore at different quencher concentrations. A considerable bathochromic shift was observed in the fluorescence wavelength compared to the absorption wavelength as we go from a high polar solvent, *i.e*., 100% ACN, to a low polar solvent, *i.e*., 100% DXN. This is attributed to the dipole–dipole interaction between the solute and the solvent molecules in the excited state than in the ground state.

**Table 1:** Fluorescence Intensity (I) of [2AHBC] as a Function of Quencher (Aniline) Concentration [Q] at a Fixed Solute Concentration (1 × 10^–6^ M) in Different ACN + DXN Solvent Mixtures at Room Temperature

	100% ACN + 0% DXN λ_exi_ = 379 nm, λ_emi_ = 456 nm		80% CAN + 20% DXN λ_exi_ = 375 nm, λ_emi_ = 450 nm		60% CAN + 40% DXN λ_exi_ = 375 nm, λ_emi_ = 447 nm		40% CAN + 60% DXN λ_exi_ = 375 nm, λ_emi_ = 443 nm		20% ACN + 80% DXN λ_exi_ = 375 nm, λ_emi_ = 440 nm		0% CAN + 100% DXN λ_exi_ = 380 nm, λ_emi_ = 420.5 nm	
[*Q*]	*I*	*I*_0_/*I*	*I*	*I*_0_/*I*	*I*	*I*_0_/*I*	*I*	*I*_0_/*I*	*I*	*I*_0_/*I*	*I*	*I*_0_/*I*
0.00	4665.500		3747.270		3600.000		3560.000		3500.000		3028.000	
0.02	3301.952	1.412	2867.341	1.306	2873.643	1.252	2768.762	1.285	2892.044	1.210	2564.158	1.180
0.04	2421.604	1.926	2184.928	1.715	2329.616	1.545	2160.823	1.647	2389.436	1.464	2144.113	1.412
0.06	1818.371	2.565	1669.565	2.244	1891.168	1.903	1674.593	2.125	1959.627	1.786	1760.470	1.719
0.08	1394.835	3.344	1229.971	3.046	1558.597	2.312	1278.738	2.783	1587.816	2.204	1410.314	2.147
0.10	1069.995	4.360	963.880	3.887	1232.003	2.922	929.138	3.831	1253.798	2.791	1084.714	2.791

**Figure 4:**
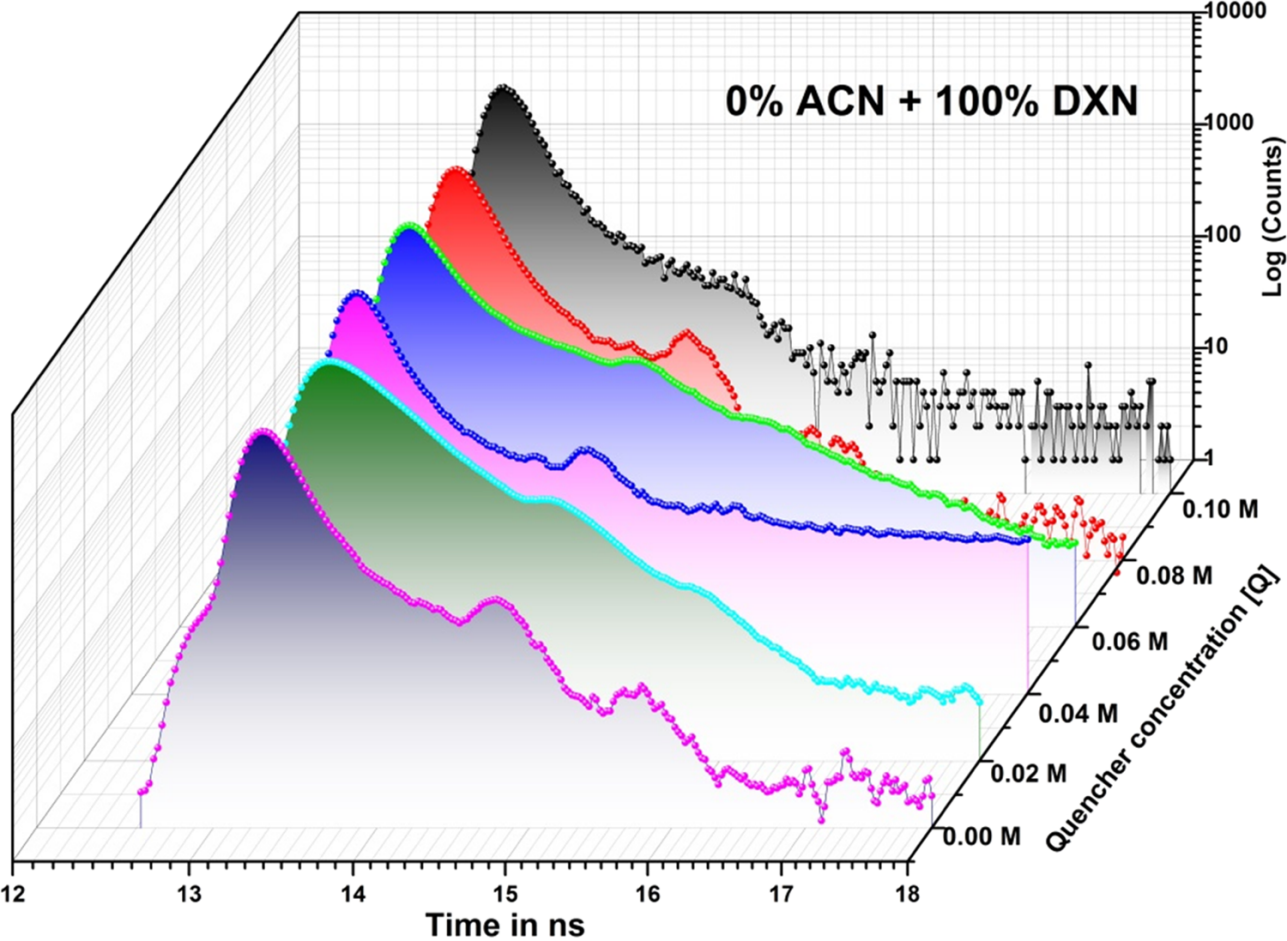
Fluorescence decay curves of 2AHBC + with and without aniline in 0% ACN + 100% DXN.

However, as seen in Figure 3, a significant decrease was observed in the intensity of the peak maxima with an increase in the aniline concentration from 0.0 to 0.10 M. This signifies that aniline successfully quenches the fluorescence intensity of the solute molecule 2AHBC. However, the lifetime of 2AHBC was recorded by varying the quencher concentration in 100% DXN solvent. An increase in the quencher concentration was observed with a decrease in the lifetime of the solute under study.^33,34^Figure 4 shows the transient state lifetime decay profile of 2AHBC in 0% ACN + 100% DXN with and without a quencher at room temperature. The lifetime of 2AHBC was biexponential without the quencher and was found to be τ_0_ = 1.10 ns.

### 3.2 Stern–Volmer (S-V) Plots

**Figure 5:**
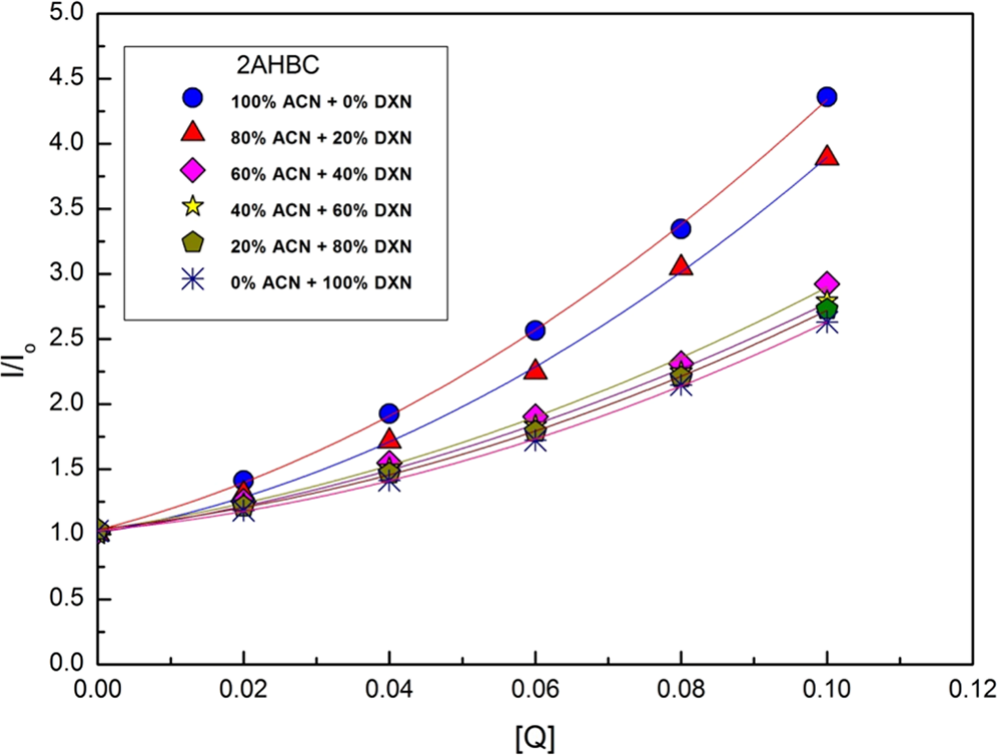
Steady-state S-V plots for 2AHBC + different concentrations of aniline in various solvent mixtures.

**Figure 6:**
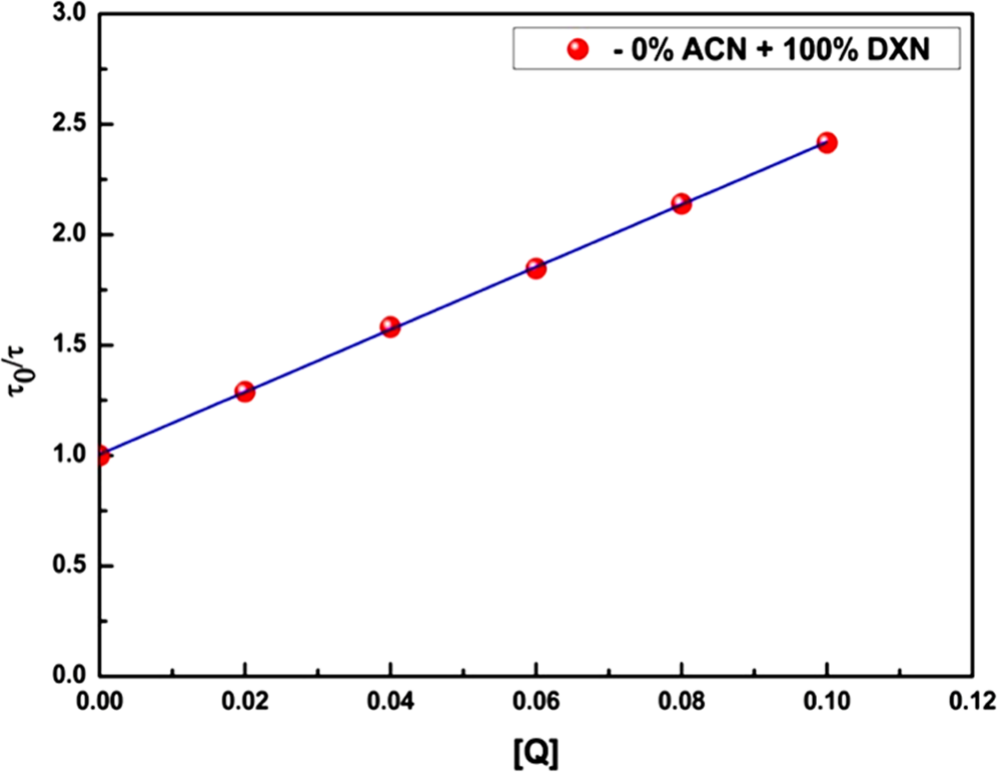
Transient state S-V plots for 2AHBC + different concentration of aniline in 0% ACN + 100% DXN.

The behavior of the solute molecule under the influence of binary solvent mixtures on the fluorescence quenching mechanism has been studied with the help of S-V relations. With the help of eqs 1 and 2, S-V plots were constructed in the steady state and in the transient state,^35^ respectively, and are shown in Figures 5 and 6.
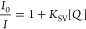
1
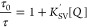
2where *K*_SV_ = *k*_q_τ_0_ called the Stern–Volmer constant, *k*_q_ is the bimolecular quenching rate constant, and τ_0_ represents the lifetime of the excited solute molecule in the absence of quencher [*Q*].

In the present case, the S-V plot in the steady state shows the concave upward curve in all ACN + DXN solvent mixtures.^36^ The presence of static quenching may be the reason for the presence of static quenching. By comparing Figures 5 and 6, we can say that both types of quenching, static and dynamic quenching, are present. The pronounced difference in transient-state and steady-state measurements also shows that the static processes are strongly dominating in the quenching of 2AHBC fluorescence by the aniline used in this work. However, the S-V plot in the transient method shows linearity^37^ in ACN + DXN solvent mixtures. It signifies that the presence of static quenching is either due to the sphere of action model or the formation of the ground-state complex.^38−40^

**Figure 7:**
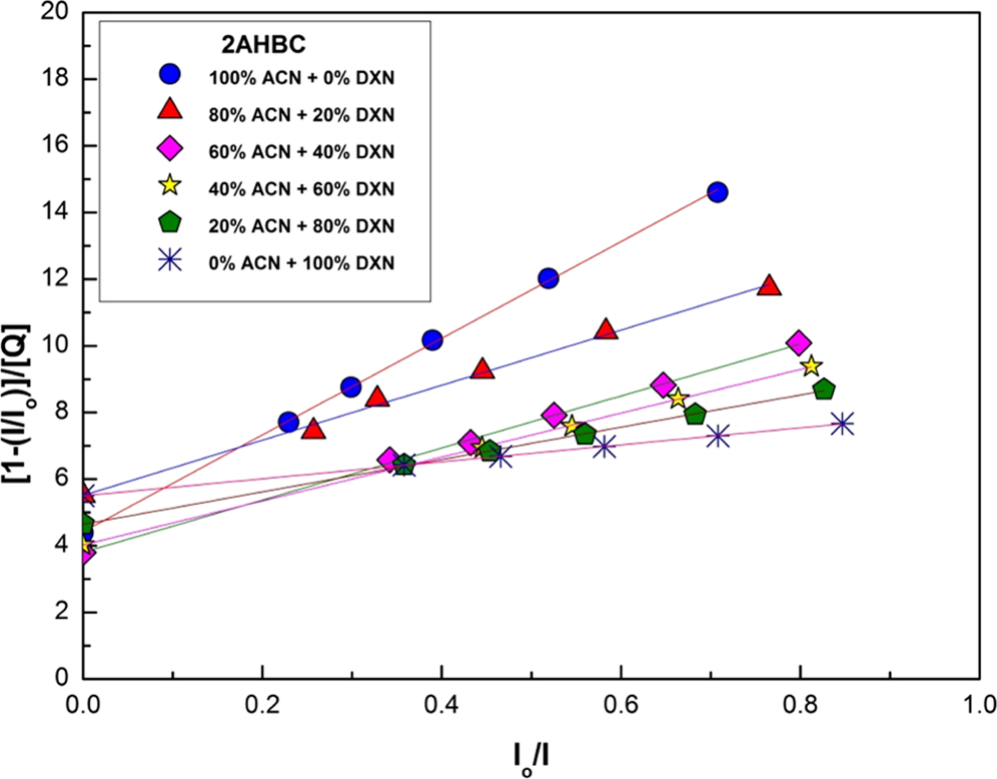
Modified S-V plot for 2AHBC in binary solvent mixtures.

According to the modified Stern–Volmer equation, S-V plots were constructed in the steady state and are shown in Figure 7
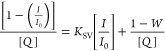
3where *W* represents the partially quenched particles, 1 – *W* represents the unquenched particles, *I*_0_ is the intensity of the solute without a quencher molecule, and *I* is the intensity of the solute with a quencher molecule. The S-V quenching constant (*K*_SV_) and a portion of the quenched particles (*W*) can be easily determined from eq 3.^41^ However, the kinetic distance (*r*) and volume of the static quenching constant (*V*) are evaluated by the relation .

### 3.3 Ground-State Complex Model

The nonlinear behavior of the S-V plot can be analyzed by incorporating eq 4
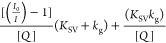
4where *k*_g_ represents the association constant.

This model can also be applied when there is any considerable change in the absorption or emission spectrum of the solute.^42^ As we have observed a slight bathochromic shift in absorption spectra and considerable bathochromic shift in emission spectra, we applied the ground-state complex model using spectral data. But, unfortunately, we have obtained all *k*_g_ values as imaginary. Hence, we can suggest that the ground-state complex model does not hold well for the present molecule in all ACN + DXN solvent mixtures.^18^

### 3.4 Sphere of Action Model

The bimolecular parameters and quenching rate constants were determined by a modified S-V plot for 2AHBC. Table 2 presents the dielectric constant of solvent mixtures and quenching rate constants. The bottom of the table contains the radii of the solute (*R*_S_) and quencher (*R*_Q_). The obtained results were correlated with the literature.^40−43^ It is observed that the calculated values of encounter distance (*R*) are almost double the kinetic distance (*r*) in all of the binary solvent mixtures. This demonstrates that the present work obeys the sphere of action model^44^ and hence confirms the presence of static quenching reactions. However, static effects can also be evident from diffusion-limited reactions.^45^ Further, the concave upward curve from the linearity in the S-V plot signifies the simultaneous occurrence of static and dynamic quenching.^46^

**Table 2:** Various Fluorescence Quenching Parameters of [2AHBC] in ACN + DXN Solvent Mixtures

solvent mixture (% v/v)	ε^a^	*K*_SV_^b^ (M^–1^)	(*k*_q_ × 10^–9^)^c^ (M^–1^ s^–1^)	[(1 – *w*)/*Q*]^dI^	(*W*)^e^	(*V*)^f^ (M^–1^)	(*r*)^g^ (Å)
100% ACN + 0% DXN	36.000	14.516	13.196	4.420	0.558–0.912	6.119	13.430
80% ACN + 20% DXN	28.600	8.274	7.522	5.509	0.449–0.889	8.499	14.990
60% ACN + 40% DXN	22.000	7.820	7.109	3.804	0.619–0.924	4.985	12.550
40% ACN + 60% DXN	15.300	7.248	6.589	5.445	0.455–0.891	8.340	14.890
20% ACN + 80% DXN	8.100	4.852	4.411	4.644	0.535–0.907	6.563	13.750
0% ACN + 100% DXN	2.100	2.545	2.314	5.498	0.450–0.890	8.472	14.970

a Dielectric constant of ACN + DXN solvent mixture.

b S-V quenching constant.

c Bimolecular quenching rate constant.

dI ntercept.

e Unquenched complex.

f Static quenching constant.

g Kinetic distance.

### 3.5 Finite Sink Approximation Model

In the case of static quenching, diffusion-limited quenching reactions are confirmed by applying eq 5.^47−49^
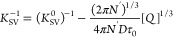
5where *K*_SV_^0^ = 4π*N*′ *RD*τ_0_*k*_a_/(4π*N*′ *RD* + *k*_a_). Using slopes and intercept of the linear plot of *K*_SV_^–1^ against [*Q*]^1/3^, the value of *K*_SV_^0^ at [*Q*] = 0 and diffusion coefficient *D* were determined. *K*_SV_^0^ can also be written as
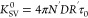
6The distance parameter (*R*′) is evaluated using eq 7 as given below
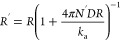
7Quenching reactions were diffusion-limited,^49^ provided *k*_a_ > *k*_d_, *i.e*., for *R*′ < *R*, and *k*_q_ > 4π*N*′*R*′ *D* for *R*′ > *R.*(32)

**Figure 8:**
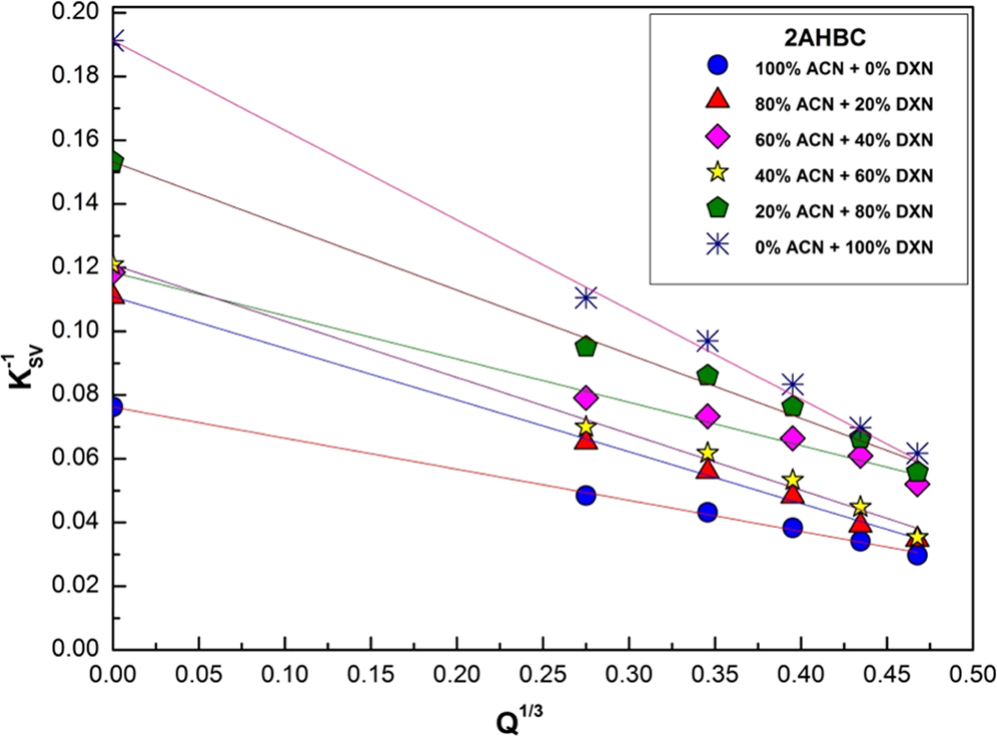
Plots of *K*_SV_^–1^ as a function of [*Q*]^1/3^ for 2AHBC.

The linear plots of *K*_SV_^–1^as a function of [*Q*]^1/3^ for 2AHBC is shown in Figure 8. Table 3 presents the values of *K*_SV_^0^, *D*, and distance parameter (*R*′) evaluated from eq 7. The obtained results were compared and found to be consistent with the literature.^50,51^ However, according to Zeng and Joshi^45,46^ et al., quenching reactions are said to be diffusion-limited only when *R*′ is larger than *R*(52,53) and *k*_q_*>* 4π*N*′*R*′*D*.^54,55^ From Table 3, it is confirmed that *R*′ *> R* and *k*_q_*>* 4π*N*′*R*′*D*^56,57^ in all of the ACN + DXN solvent mixtures. Hence, reactions are said to be diffusion-limited.

**Table 3:** Steady-State S-V Constant at [*Q*] = 0 and Other Quenching Constants for [2AHBC]^a^^,^^b^

binary solvent mixture (% v/v)	*K*_SV_^0^ (M^–1^)	*D*^b^ = *D* × 10^5^ (cm^2^ s^–1^)	*R*′ (Å)	4π*N*′*DR*′ × 10^–9^ or *k*_d_ (M^–1^ s^–1^)	*k*_a_ × 10^–9^ (M^–1^ s^–1^)	*k*_q_ × 10^–9^ (M^–1^ s^–1^)	1/η (10^–2^ × P^–1^)
100% ACN + 0% DXN	13.119	1.918	8.220	11.926		23.345	2.904
80% ACN + 20% DXN	9.009	1.155	9.370	8.190		18.552	2.871
60% ACN + 40% DXN	8.436	1.376	7.330	7.639		13.297	2.439
40% ACN + 60% DXN	8.268	1.058	9.390	7.516		16.443	1.881
20% ACN + 80% DXN	6.536	0.931	8.430	5.942		11.570	1.295
0% ACN + 100% DXN	5.228	0.666	9.430	4.752		10.515	0.848

a (2AHBC) *R* = *R*_S_ (3.68 Å) + *R*_Q_ (2.84 Å) = 6.52 Å. (*R*_S,_ radii of the solute, *R*_Q_ radii of the quencher).

b τ_0_ = 1.10 ns, *D*^b^: diffusion coefficients determined from the finite sink model.

Using eqs 6 and 7, the distance parameter (*R*) and the mutual diffusion coefficient (*D*) were determined and are presented in Table 4. It was observed that there was no similarity between *D*^a^ and *D*^b^ and *R*′ and *R* in all of the ACN + DXN solvent mixtures. This is due to the deviations in the values of the adjustable parameter “*a*” in the Stokes–Einstein relation and the approximated values of the atomic volume in the Edward′s empirical relation.^53^ A similar discrepancy was observed in other studies.^58−60^ Hence, we can conclude that the finite sink approximation model helped us only to extract *D* and *R*′ values.

**Table 4:** Mutual Diffusion Coefficients and Distance Parameter *R*′ for [2AHBC]^a^^,^^b^

solvent mixture (% v/v)	*D*^a^ = *D* × 10^5^ (cm^2^ s^–1^)	*D*^b^ = *D* × 10^5^ (cm^2^ s^–1^)	*R*′ (Å)
100% ACN + 0% DXN	6.304	1.918	8.220
80% ACN + 20% DXN	6.155	1.155	9.370
60% ACN + 40% DXN	5.229	1.376	7.330
40% ACN + 60% DXN	4.032	1.058	9.390
20% ACN + 80% DXN	2.776	0.931	8.430
0% ACN + 100% DXN	1.564	0.666	9.430

a *D*^a^: diffusion coefficients by the Stokes–Einstein relation. *D*^b^: diffusion coefficients by the finite sink model.

b (2AHBC) *R* = 6.52 Å.

**Figure 9:**
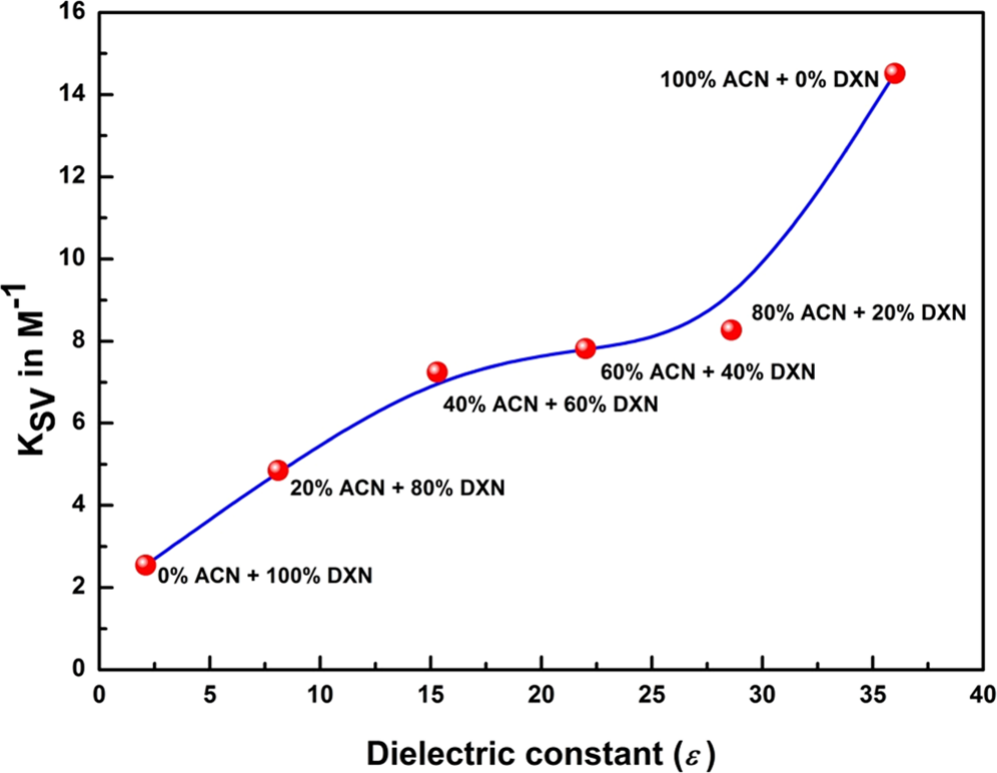
Plot of *K*_SV_ against the dielectric constant (ε) for 2AHBC.

Furthermore, the effect of the dielectric constant of the solvent mixtures on the quenching constant of 2AHBC was studied. Figure 9 shows the nonlinear plot of *K*_SV_ against the dielectric constant (ε) for 2AHBC. It was observed that with an increase in the dielectric constant of the solvent mixture, there was an increase in *K*_SV_, which shows the improved reaction rate and charge transfer nature. As we continue to increase the polar nature of the solvent, the dielectric constant increases, which in turn stabilizes the reacting species. The fluorescence quenching will be more pronounced for polar solvents with increasing dielectric constants, which might be due to the effect of the hydrogen bond on a radiationless deactivation process. This indicates an elevated charge transfer nature of the exciplex in the ACN + DXN solvent mixtures. Similar results were reported in other literature studies.^58,59^

**Figure 10:**
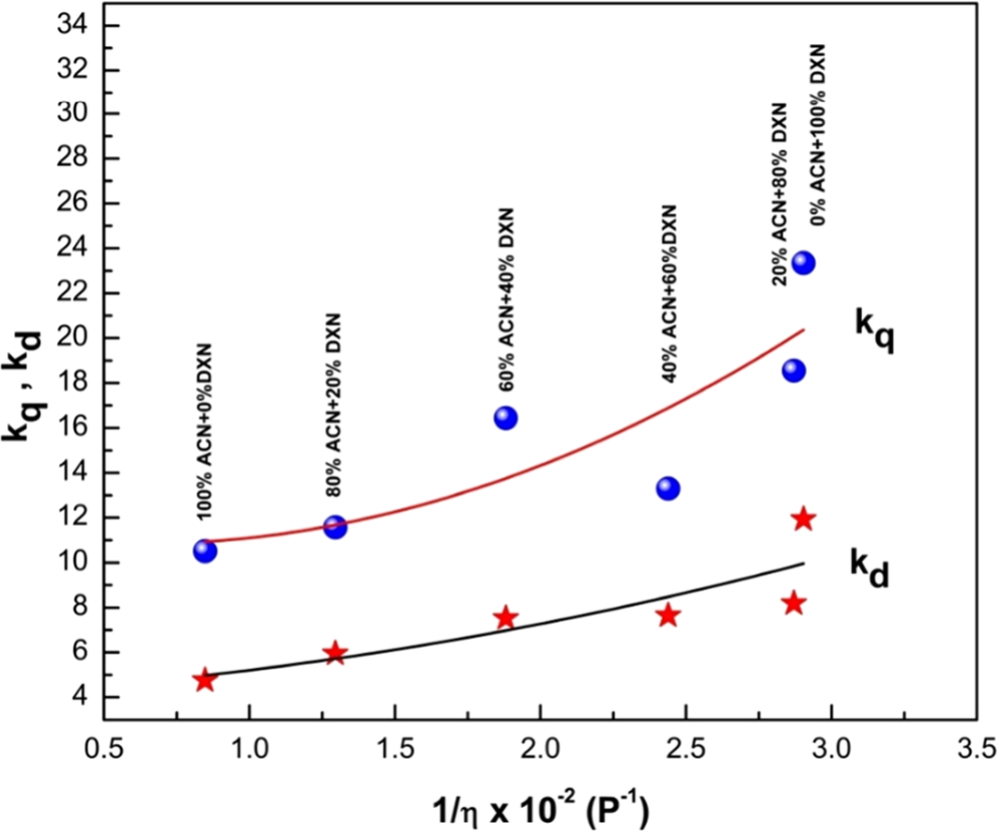
Plot of *k*_q_, *k*_d_ against 1/η × 10^–2^ of the binary solvent mixture (ACN + DXN) for 2AHBC.

**Figure 11:**
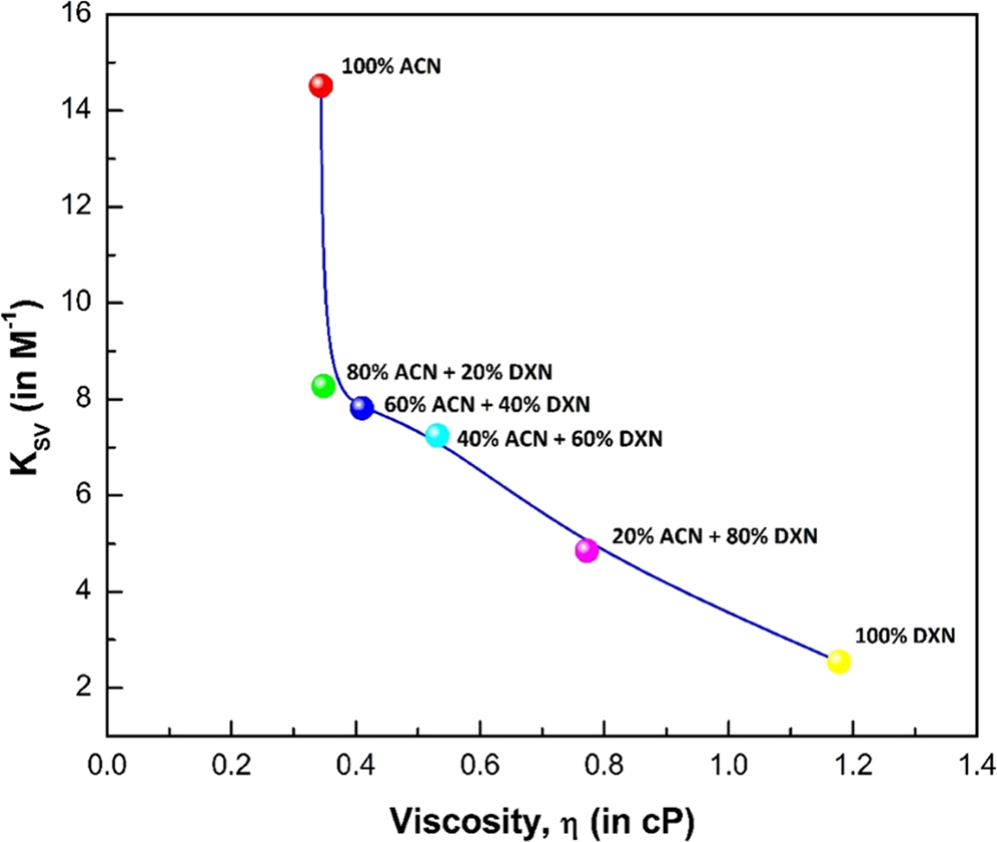
Plot of *K*_SV_ against viscosity of the binary solvent mixture (ACN + DXN) (η) for 2AHBC.

Figure 10 shows the graph of the frequency of encounter (*k*_d_) against the inverse viscosity of different binary mixtures. As seen in Figure 11, both the frequency of encounter, *k*_d_, and quenching rate parameter, *k*_q_, increase with a decrease in viscosity. Hence, we can conclude that quenching is controlled by material diffusion. As shown in Figure 11, the dependency of the bimolecular quenching rate parameter (*K*_SV_) was correlated with the viscosity of the binary solvent mixture. For the present molecule, an inverse dependency of viscosity of solvent mixture on *K*_SV_ was observed.^51^ The fluorescence quenching has no major impact or a less pronounced impact on the viscosity of solvents. This is attributed to the mechanisms such as single–triplet conversion, charge transfer complex, and chemical reactions that might play a role.

### 3.6 Antibiotic-Resistant Target in *Pseudomonas aeruginosa*

**Figure 12:**
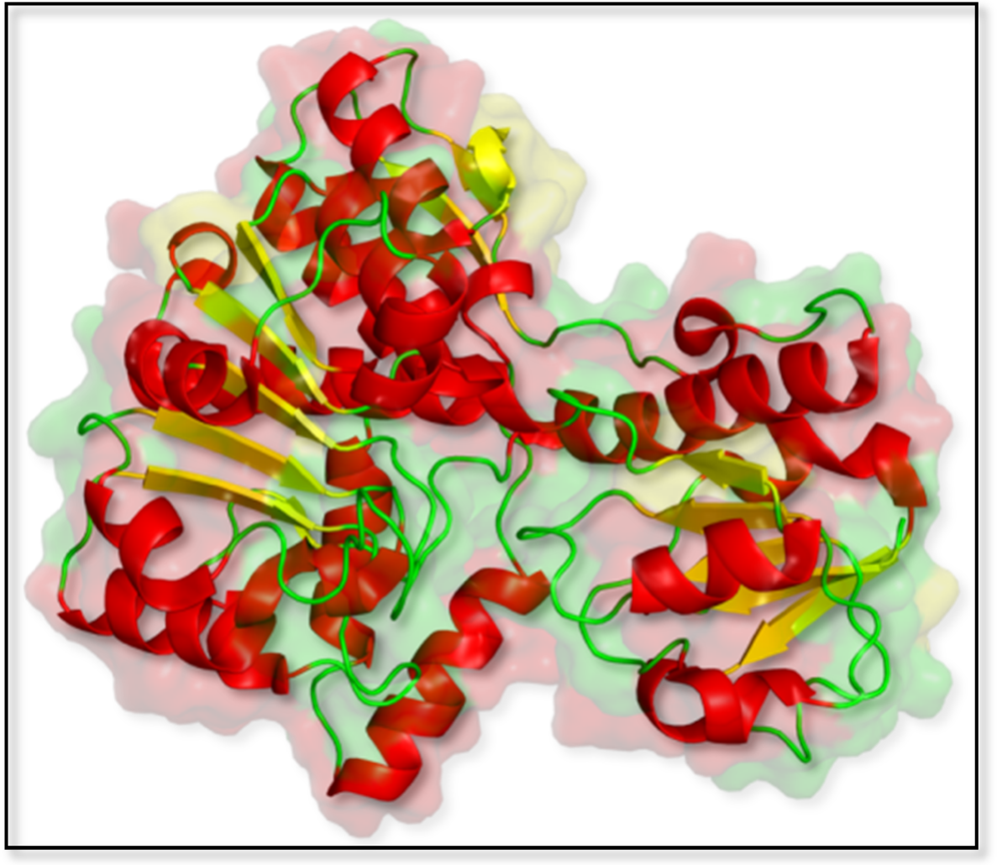
3D structure of erythronate-4-phosphate dehydrogenase structure from *P. aeruginosa* (PDB id: 2O4C) colored based on the secondary structure (helix: red, sheet: yellow, loop: green).

The analysis of the *P. aeruginosa* genome for the possible antibiotic-resistant targets was carried out using ARTS (Antibiotic-Resistant Target Seeker V2). The analyzed genome showed that the genome of *P. aeruginosa* consists of a total of 5681 genes, out of which 584 genes are core genes or the essential genes, 13 genes are part of biosynthetic pathway gene clusters, and 63 genes are antibiotic-resistant models. Erythronate-4-phosphate dehydrogenase is one of the enzymes that are an essential protein in the lifecycle of *P. aeruginosa* and is also part of the antibiotic-resistant model. The three-dimensional (3D) crystallized structure of erythronate-4-phosphate dehydrogenase was obtained from the PDB (Protein Data Bank (PDB) id: 2O4C).^61^Figure 12 shows the 3D structure of erythronate-4-phosphate dehydrogenase with a resolution of 2.30 Å.

#### 3.6.1 Molecular Docking

Molecular docking was carried out on erythronate-4-phosphate dehydrogenase with compound 2AHBC (Pubchem id: 748172) to find the best possible mode of binding. Details of ADMET properties of the compound 2-acetyl-3*H*-benzo[*f*]chromen-3-one are presented in Table S2. ADMET analysis of the compound 2AHBC was carried out, and it was seen that the compound passed Lipinski’s rule of drug-likeness. It was also predicted that the compound has high GI absorption and is also BBB permeate. The best fit was selected based on the binding affinity score and based on the interactions with the important residues of erythronate-4-phosphate dehydrogenase. The target protein structure was further cleaned by removing the waters using Autodock tools, and the cocrystallized ligand of NAD+ was further removed for the molecular docking. The binding space of the cocrystallized was analyzed, and the key residues were identified. A total of 103 water molecules were removed from the structure.^62^

**Figure 13:**
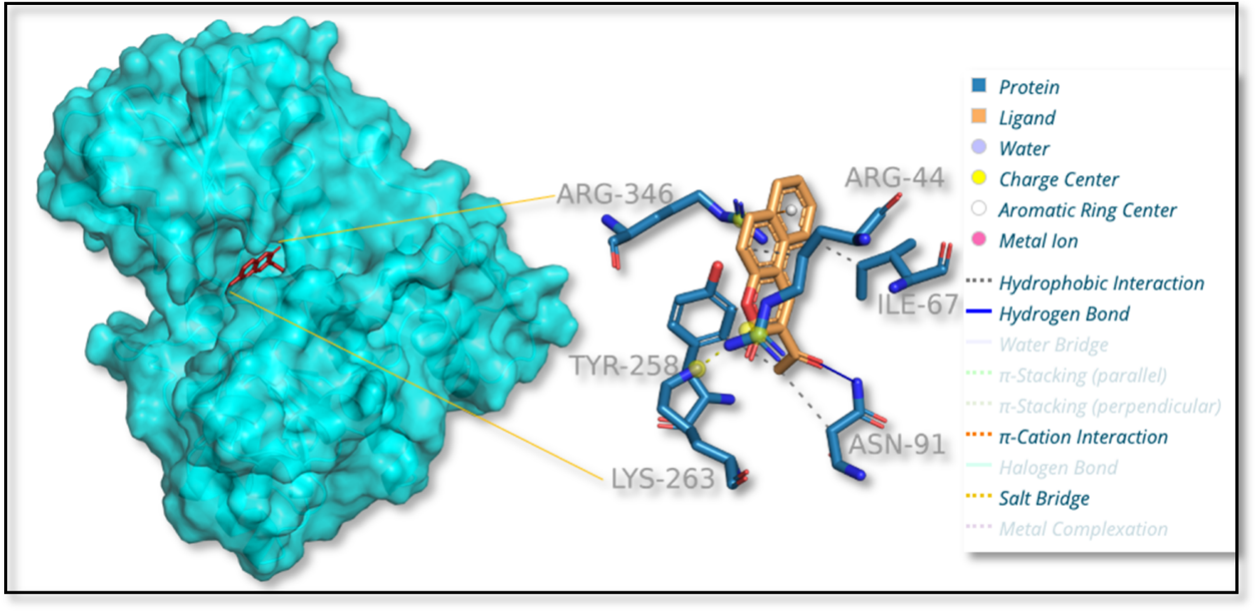
3D image of the protein–ligand complex of erythronate-4-phosphate dehydrogenase with 2AHBC with interacting sites.

The grid box for the docking was considered based on the coordinates of the cocrystallized ligand. The coordinates were *x* = 27.37, *y* = 85.45, and *z* = 2.32, and the size of the grid box for the docking was 25 points for *x*, *y*, and *z* coordinates. The docking of erythronate-4-phosphate dehydrogenase with the optimized structure of 2AHBC was carried out with exhaustiveness of 100 (high accuracy), and the binding affinity was determined to be −7.9 kcal mol^–1^. A comprehensive analysis of the complex was done, and the involvement of key amino acid residues in the active site is tabulated in Table 5 and Figure 13.

**Table 5:** Docking Result of Erythronate-4-phosphate Dehydrogenase with 2-Acetyl-3*H*-benzo[*f*]chromen-3-one with Interaction Sites

compound name	docking score (Kcal mol^–1^)	interaction residues (hydrogen bond)	interaction residues (hydrophobic interactions)	pi-cation interactions
2-acetyl-3*H*-benzo[*f*]chromen-3-one	–7.9	ASN91	ARG44, ILE67, ASN91, TYR258	ARG346

### 3.7 Antimicrobial Evaluation of 2-Acetyl-3*H*-benzo[*f*]chromen-3-one

**Figure 14:**
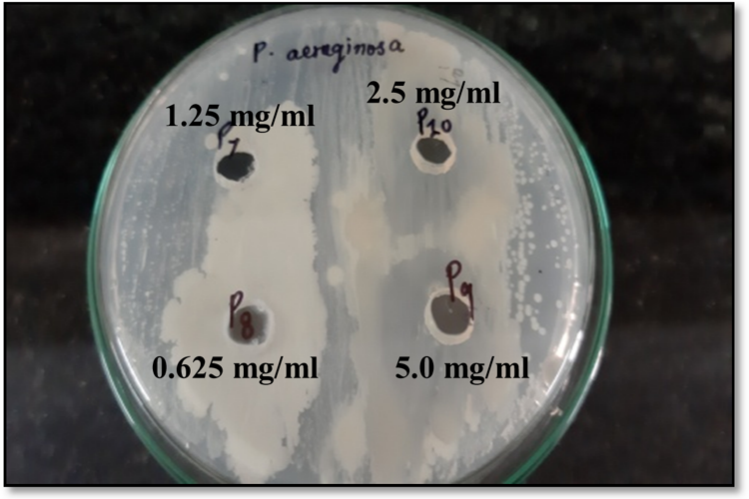
Minimum inhibitory concentration of 2AHBC against *P. aeruginosa*.

*In vitro* antimicrobial attributes of 2AHBC are presented in Table 6. Based on the antibacterial evaluation by the agar well diffusion method, the 2AHBC compound showed a diverse inhibitory effect on different pathogens. The compound was revealed to exhibit antibacterial attributes against all of the evaluated microbial strains. A zone of inhibition greater than 10 mm was termed a prominent effect. A pronounced effect was observed against *P. Aeruginosa* (23.3 ± 0.9 mm) (Gram-negative bacteria) and *B. subtilis* (19.5 ± 0.8 mm) (Gram-positive bacteria). *In vitro* antifungal activity of 2AHBC against *C. albicans* was also promising, with a 16.7 ± 1.3 mm zone of inhibition based on the analysis of the agar disk diffusion method. No effect with DMSO (negative control) and inhibitory effect with standard gentamicin (positive control) were observed. Evaluation of the MIC of the compound against *P. aeruginosa* was carried out considering the highest inhibitory effect shown on the pathogen. *P. aeruginosa* was found to be susceptible, exhibiting significant inhibition with a MIC of 2.5 mg mL^–1^ (Figure 14). Bacterial infections have led to significant health disasters globally possibly due to drug resistance. As a result, this has led to the development of new prospective drug molecules with both a specific and broad range of antimicrobial effects. The Gram-negative microbial strains are usually resistant to drugs due to the effective outer membrane that is constructed of lipopolysaccharide, which restricts the penetration of the drug molecules. It also recognizes and pumps out the chemical molecules out of the cell without allowing them to interact with the drug target. Hence, designing and synthesizing a drug molecule specific for Gram-negative microbial strain is crucial. Coumarins are desirable molecules for the development of novel antibacterial agents.^63^ Several synthesized coumarins are reported in the literature for various biological activities. El-Wahab et al.^64^ reported the synthesis of 2-(heteroaryl)-3*H*-benzo[*f*]chromen-3-ones with antibacterial potential against *E. coli* and *S. aureus*. An application of antimicrobial polyurethane coating using 2-(2-amino-1,3-thiazol-4-yl)-3*H*-benzo[*f*]chromen-3-one (a coumarin thiazole derivative) possessing antimicrobial potential has been reported by El-Wahab et al.^64^ Raj et al.^65^ synthesized several compounds and studied the antimicrobial effect and MIC against several bacterial and fungal pathogens, but none of the compounds could inhibit the growth of *P. aeruginosa*, and interestingly, we report the inhibition of *P. aeruginosa* by our synthesized compound. Studies showed that the electron-withdrawing groups usually exhibit the antifungal activity of the compounds against *C. albicans* and *Candida tropicalis*. The electron-releasing group may possibly promote the antibacterial effect against Gram-positive pathogens, and electron-withdrawing groups may trigger antibacterial effects against both Gram-positive and Gram-negative microbial strains^65^ as reported for newly synthesized 2*H*-benzo[*h*] chromene derivatives as a group of antibacterial adjuvants that exhibit effective biological properties. However, not much literature is available on the antimicrobial properties of the compound 2-acetyl-3*H*-benzo[*f*]chromen-3-one. Each data value represents the mean±SD value of triplicate experiments and is significant at *p* < 0.05.

**Table 6:** Antimicrobial Properties of 2-acetyl-3*H*-benzo[*f*]chromen-3-one

	pathogens	zone of inhibition (mm ± SD)
gram negative bacterial cultures	*Klebsiellapneumoniae* (MTCC 109)	07.2 ± 0.5
	*Salmonella typhimurium* (MTCC 98)	11.4 ± 0.6
	*aepPseudomonas aeruginosa* (MTCC 2297)	23.3 ± 0.9
	*Escherichia coli* (MTCC 443)	16.8 ± 0.8
gram positive bacterial cultures	*Micrococcus luteus* (NCIM 2871)	15.2 ± 1.1
	*Bacillus cereus* (NCIM 2217)	17.4 ± 0.7
	*Bacillus subtilis* (NCIM 2718)	19.5 ± 0.8
	*Staphylococcus aureus* (MTCC 737)	12.2 ± 1.2
fungal culture	*Candida albicans*	16.7 ± 1.3

## 4 Conclusions

Fluorescence quenching of 2AHBC in solvent mixtures of ACN + DXN was analyzed. The Stern–Volmer plot showed a concave upward curvature in the presence of quenchers. The studied system obeys the sphere of action static quenching model and confirms the presence of static quenching. For 2AHBC, *k*_q_ was found to be greater than 4π*N*′*R*′*D*. Hence, quenching reactions were found to be diffusion-limited. There was a nonlinear dependency on the dielectric constant of the binary solvent mixture, and *K*_SV_ suggests high charge transfer of the exciplex in the binary solvent mixture and an inverse dependency of viscosity of solvent mixture on *K*_SV_. The fluorescence quenching is more efficient in polar solvents, and in them, quenching decreases as the viscosity of the medium increases. This is attributed to the mechanisms such as single–triplet conversion, charge transfer complex, and chemical reactions that might play a role. We also expect fluorescence quenching to find its application in studying other biological processes, such as RNA folding or conformational changes of enzymes during functioning. In view of this, the fluorophore was first applied for druglike activity, and then it was checked for antimicrobial activity through bioinformatics tools, which showed positive results. Docking studies were performed for its antimicrobial activity against a facultative pathogen *P. aeruginosa*, as it is multi-drug-resistant. We have selected erythronate-4-phosphate dehydrogenase, which is part of the antibiotic resistance mechanism. After positive *in silico* studies, in vitro studies were carried out with selected pathogens to prove their antimicrobial activity, and the results were encouraging, with a higher zone of clearance for *P. aeruginosa* compared to other pathogens in the study. These considerations can strongly indicate that fluorescence quenching mechanisms are useful and extensively appropriate to obtain enriched information about the structure and dynamics of biologically important macromolecular systems.

## Supporting Information Available

Details of numerous applications of fluorescence spectroscopy (Table S1) and details of ADMET properties of the compound 2-acetyl-3*H*-benzo[*f*]chromen-3-one (Table S2) (PDF)
