# Supplementary material for: Investigation of the Fluorescence Turn-off Mechanism, Genome, Molecular Docking In Silico and In Vitro Studies of 2-Acetyl-3H-benzo[f]chromen-3-one
Source: ACS Omega. 2022 Jun 29;7(27):23759–70. doi: 10.1021/acsomega.2c02424 (PMC9280945; doi:10.1021/acsomega.2c02424)
Supplement: Supplementary file 1 — ao2c02424_si_001.pdf [file ao2c02424_si_001.pdf]

Investigation on Fluorescence turn-off mechanism, Genome, Molecular docking *in silico* and *in vitro* studies of 2-acetyl-3H-benzo[f]chromen-3-one

Varsha V Koppal[a], Raveendra Melavanki[b], Raviraj Kusanur[c], Zabin K Bagewadi[d*], Deepak A Yaraguppi[d], Sanjay H Deshpande[d] and Ninganagouda R Patil[e]

[a]Department of Physics, KLE Technological University, Hubli 580031, Karnataka, India

[b]Department of Physics, M S Ramaiah Institute of Technology, Bangalore 560054, Karnataka, India

[c]Department of Chemistry, R.V. College of Engineering, Bangalore 560059, Karnataka, India

[d]Department of Biotechnology, KLE Technological University, Hubballi, Karnataka 580031, India

[e]Department of Physics, B V B College of Engineering and Technology, Hubli 580031, Karnataka, India

**Corresponding Authors E-mails: Ninganagouda R Patil: patilnr23@gmail.com; *Zabin K Bagewadi: zabinb@gmail.com

Supplementary Information

Table S1: Some biological and medical applications of fluorescence spectroscopy

**Application**	Technique
Conformation and dynamics of macromolecules (proteins and nucleic acids)	
**Molecular weight of macromolecules**	Polarized fluorescence, FRAP
**Shape of macromolecules**	Polarized fluorescence, FRAP, excitation energy transfer
**Flexibility of macromolecules**	Polarized fluorescence
**Distance between functional sites on a macromolecule**	Excitation energy transfer
**Dynamic polarity of functional sites**	Polarity sensitive probes
Conformation and dynamics of supramolecular assemblies	
**Cytoskeleton**	Polarized fluorescence, Excitation energy transfer
**Ribosomes**	Polarized fluorescence, Excitation energy transfer
**Chromatin**	Polarized fluorescence
Conformation and dynamics of biological membranes	
**Lateral mobility of membrane**	FRAP

**receptors**	
**Fluidity of biological membranes**	FRAP, fluorescence polarization, excimer formation
**Spatial orientation of membrane components**	Polarized fluorescence
**Proximity of functional sites**	Excitation energy transfer
Clinical applications	
**Immunoassays**	Time discriminated fluorescence intensity
**DNA probe assays**	Time discriminated fluorescence intensity
**Measurement of arterial blood Po_2, Pco_2, pH**	Fluorescence intensity or lifetime measurements with long-lived fluorophores

**Table S2: ADMET properties of the compound**	
**Molecule**	**2-acetyl-3-Acetylbenzo[f]coumarin (PubChem id:748172)**
Canonical SMILES	CC(=O)c1cc2c(oc1=O)ccc1c2cccc1
Formula	$C_{15}H_{10}O_3$
MW	238.24
#Heavy atoms	18
#Aromatic heavy atoms	14
Fraction Csp3	0.07
#Rotatable bonds	1
#H-bond acceptors	3
#H-bond donors	0
MR	70.18
TPSA	47.28
iLOGP	1.82
XLOGP3	3.05
WLOGP	3.15
MLOGP	2.17

Silicos-IT Log P	3.84
Consensus Log P	2.8
ESOL Log S	-3.75
ESOL Solubility (mg/ml)	4.25E-02
ESOL Solubility (mol/l)	1.79E-04
ESOL Class	Soluble
Ali Log S	-3.71
Ali Solubility (mg/ml)	4.66E-02
Ali Solubility (mol/l)	1.95E-04
Ali Class	Soluble
Silicos-IT LogSw	-5.63
Silicos-IT Solubility (mg/ml)	5.60E-04
Silicos-IT Solubility (mol/l)	2.35E-06
Silicos-IT class	Moderately soluble
GI absorption	High
BBB permeant	Yes
Pgp substrate	No
CYP1A2 inhibitor	Yes
CYP2C19 inhibitor	Yes
CYP2C9 inhibitor	No
CYP2D6 inhibitor	No
CYP3A4 inhibitor	No
log Kp (cm/s)	-5.59
Lipinski #violations	0
Ghose #violations	0
Veber #violations	0
Egan #violations	0
Muegge #violations	0
Bioavailability Score	0.55
PAINS #alerts	0

Brenk #alerts	2
Leadlikeness #violations	1
Synthetic Accessibility	2.83
